# Tofacitinib in the treatment of ulcerative colitis: A position paper issued by the Inflammatory Bowel Disease Working Group of the Austrian Society of Gastroenterology and Hepatology (ÖGGH)

**DOI:** 10.1007/s00508-022-02110-2

**Published:** 2022-12-01

**Authors:** Maximilian Kutschera, Gottfried Novacek, Walter Reinisch, Christoph Högenauer, Wolfgang Petritsch, Thomas Haas, Alexander Moschen, Clemens Dejaco

**Affiliations:** 1 Division of Gastroenterology and Hepatology, Department of Internal Medicine III, Division of Gastroenterology and Hepatology, Medical University of Vienna, Vienna, Austria; 2 Division of Gastroenterology and Hepatology, Medical Department, Medical University of Graz, Graz, Austria; 3 Gastroenterology Office (Darmpraxis), Salzburg, Austria; 4 Second Medical Department, Kepler University Hospital, Linz, Austria

**Keywords:** Ulcerative colitis, Small molecules, Janus kinase inhibitor, Safety, Effectiveness

## Abstract

Ulcerative colitis (UC) is one of the main forms of inflammatory bowel disease (IBD). Despite the widening range of drug treatment options, primary nonresponse, secondary loss of response as well as adverse events call for additional treatment alternatives.

Tofacitinib is an oral small-molecule drug of the class of Janus kinase inhibitors which, in the European Union, was approved for the treatment of moderate to severe active UC in August 2018. This position paper, drawn up by the IBD Working Group of the Austrian Society of Gastroenterology and Hepatology, summarizes the mechanism of action, clinical development, marketing authorization status, efficacy and safety of tofacitinib. Also, by providing a synopsis of available data from both pivotal and post-marketing studies, clinical aspects of specific interest are highlighted and discussed.

The available body of evidence indicates that tofacitinib is an additional effective medication for the treatment of UC that exhibits a good safety profile. This position paper aims at optimizing the safe and effective use of tofacitinib in daily clinical practice.

## Introduction

Ulcerative colitis (UC) is an inflammatory bowel disease, which is marked by a continuous inflammation of the colon extending from the distal sigmoid colon towards proximal and which affects the mucosa and submucosa. The objectives of treatment are to rapidly achieve and maintain steroid-free clinical and endoscopic remission.

The current treatment options include 5‑aminosalicylates, systemic and topical steroids, as well as conventional immunosuppressives and biologics, such as tumour necrosis factor (TNF) alpha inhibitors, anti-adhesion molecules and anti-interleukin (IL) 12/23 antibodies. Notwithstanding an increasing number of treatment options, the clinical problem persists that a relevant number of patients fail to show long-lasting responses to such treatment.

In the past years small molecules have been developed. Tofacitinib (Xeljanz®, Pfizer Corporation Austria, Vienna, Austria) is an orally administered small molecule of the Janus kinase (JAK) inhibitor class, which was approved in Europe in August 2018 for adults with moderately to severely active UC, who do not or no longer respond sufficiently to conventional treatment or biologics or who are intolerant to such treatment.

Tofacitinib is effective via strong and selective JAK inhibition. The JAK family comprises the intracellular tyrosine kinases (TYK) JAK1, JAK2, JAK3 and TYK2. Various cytokines bind to membrane receptors, thus leading to JAK activation and subsequently the phosphorylation and activation of signal transducer and activator of transcription (STAT) proteins. As a result, phosphorylated and dimerized STATs translocate into the cell nucleus, where they modulate gene transcription and, thus, aspects of the immune response. Tofacitinib inhibits all JAKs, especially JAK1 and JAK3, and thereby reduces the signal transduction of ILs, such as IL‑2, 4, 6, 7, 9, 15 and 21, as well as type I and type II interferons [1].

On account of this, tofacitinib displays a novel mode of action in UC treatment. Biologics inhibit cytokine signal transduction by binding and neutralizing cytokines or corresponding receptors, yet they do not reach the intracellular components of the cytokine pathways. The intervention into the JAK-STAT signalling pathway offers an alternative approach with the potential to modulate multiple cytokines by inhibiting one common signalling pathway [2].

Whether the application of tofacitinib is indicated should be decided by specialists in gastroenterology and hepatology. Apart from UC, tofacitinib was approved in Europe for the treatment of rheumatoid arthritis (RA) and psoriatic arthritis (PsA) in 2017 and 2018, respectively.

Based on the currently available data, the following position paper provides an overview of and recommendations concerning tofacitinib treatment management in UC.

## Tofacitinib in ulcerative colitis: clinical trial program

A double-blind, randomized, placebo-controlled, phase 2 dose-finding study, compared various doses (0.5, 3, 10 or 15 mg b.i. d.) with placebo over 8 weeks in 194 adults with moderate to severe UC (Mayo score ≥ 6, endoscopic Mayo subscore ≥ 2), [3]. The primary endpoint was clinical response at week 8 (see Table 1 for definitions), which was reached significantly more often among patients under 15 mg b.i. d. (78%, *p* < 0.001) and under 10 mg b.i. d. (61%, *p* = 0.1) compared to the placebo group (42%). Furthermore, patients under 15 mg b.i. d. and 10 mg b.i. d. experienced significantly more often the secondary endpoints remission, endoscopic response and endoscopic remission, than those in the placebo group. The doses of 0.5 mg b.i.d and 3 mg b.i. d. did not show a benefit over placebo neither in the primary nor in secondary endpoints.

**Table 1:** Tofacitinib in UC: definitions applied in the clinical approval studies [4]

*Remission*	Full Mayo score ≤ 2, no single subscore > 1 and rectal bleeding subscore = 0
*Mucosal healing*	Endoscopic Mayo subscore = 0 or 1
*Clinical response*	Decrease in full Mayo score of ≥ 3 points and ≥ 30% vs. baseline, which must include a decrease in the subscore for rectal bleeding of ≥ 1 point or an absolute rectal bleeding subscore = 0 or 1
*Endoscopic remission*	Endoscopic Mayo subscore = 0
*Sustained remission*	Remission at weeks 24 and 52 in OCTAVE Sustain in patients already in remission at the beginning of maintenance therapy
*Sustained corticosteroid-free remission*	Corticosteroid remission at weeks 24 and 52 in OCTAVE Sustain in patients already in remission at the beginning of maintenance therapy
*Sustained clinical response*	Clinical response at weeks 24 and 52 in OCTAVE Sustain in patients already showing clinical response at the beginning of maintenance therapy and sustained clinical response at weeks 24 and 52
*Treatment failure*	Increase in Mayo score of ≥ 3 points vs. baseline in OCTAVE Sustain, which must include an increase in the rectal bleeding subscore of ≥ 1 point and an increase in the endoscopic subscore of ≥ 1 point, i.e. an absolute endoscopic subscore of ≥ 2 following ≥ 8 weeks of treatment in OCTAVE Sustain Patients experiencing treatment failure were excluded from OCTAVE Sustain yet were eligible for participation in OCTAVE Open
*Loss of response*	Increase in partial Mayo score of ≥ 2 points vs. baseline in OCTAVE Sustain at 2 consecutive rounds, which must include an increase in the rectal bleeding subscore of ≥ 1 point vs. baseline in OCTAVE Sustain

*OCTAVE Sustain, OCTAVE Open*

**Fig. 1:**
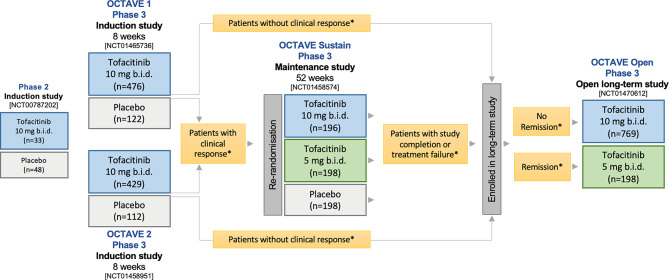
Tofacitinib in ulcerative colitis: overview of the clinical development program (for definitions, see Table 1)

Subsequently, the efficacy and safety of tofacitinib in the treatment of adult patients with moderate to severe active UC was explored in 3 randomized, double-blind, placebo-controlled phase 3 trials, the identical OCTAVE Induction 1 and OCTAVE Induction 2 studies, followed by the OCTAVE Sustain study, which recruited participants from the induction studies [5]. Patients were eligible to participate in an open, phase 3 long-term trial, OCTAVE Open, after the preceding 3 studies had been completed (Fig. 1; Table 2).

**Table 2:** Tofacitinib in ulcerative colitis: key data of the phase 3 approval studies [5]

	OCTAVE Induction 1		OCTAVE Induction 2		OCTAVE Sustain		
	Placebo	Tofacitinib 10 mg b.i. d.	Placebo	Tofacitinib 10 mg b.i. d.	Placebo	Tofacitinib 5 mg b.i. d.	Tofacitinib 10 mg b.i. d.
*Patients (n)*	122	476	112	429	198	198	197
*Duration*	8 weeks		8 weeks		52 weeks		
Primary endpoint*	Remission at week 8		Remission at week 8		Remission at week 52		
Key secondary endpoints*	Mucosal healing		Mucosal healing		Mucosal healing Sustained corticosteroid-free remission		
Other secondary endpoints*	Clinical response Endoscopic remission		Clinical response Endoscopic remission		Sustained remission Sustained clinical response Endoscopic remission		
*Oral corticosteroid application at baseline (in %)*	48	45	49	46	51	51	44
*Preceding TNF-alpha inhibitor application (in %)*	53	53	58	55	47	46	51
*Previous failure of (in %)*							
TNF-alpha inhibitor	53	51	54	52	44	42	47
Corticosteroid	80	74	74	71	76	73	76
Immunosuppressive	68	76	67	70	65	72	72

*For definitions, see Table 1

### OCTAVE Induction 1 and 2

Based on the phase 2 results, a regimen of 10 mg b.i. d. for 8 weeks was chosen for the OCTAVE Induction 1 and 2 studies. Monotherapy with tofacitinib was applied throughout the development program (i.e. without concomitant biologics or immunosuppressives, which had to be discontinued at least 8 weeks prior to study participation).

OCTAVE Induction 1 and 2 included patients, in whom at least one of the following treatments had failed or had not been tolerated: (1) oral corticosteroids, (2) immunosuppressives azathioprine or mercaptopurine or (3) TNF-alpha inhibitors infliximab or adalimumab. Concomitant administration of consistently dosed aminosalicylates and corticosteroids (maximum daily dose: 25 mg prednisone or equivalent) was admissible throughout the study [5].

### OCTAVE Sustain

Patients who had completed OCTAVE Induction 1 or 2 and who had shown clinical response after 8 weeks were eligible to participate in the OCTAVE Sustain trial. They were re-randomized to receive either tofacitinib 5 mg b.i. d., tofacitinib 10 mg b.i. d. or placebo. According to the study protocol, corticosteroids were to be tapered off within 15 weeks after joining this maintenance study. In the presence of worsened symptoms after steroid reduction, it was permissible to re-increase the steroid dosage once in the course of OCTAVE Sustain [5].

### OCTAVE Open

Patients who had failed to achieve clinical response after completing OCTAVE Induction 1 or 2, as well as those who had dropped out of OCTAVE Sustain due to treatment failure or who had completed that study according to schedule were eligible to participate in the long-term OCTAVE Open study.

Patients in remission upon entering into OCTAVE Open were treated with tofacitinib 5 mg b.i. d. and all others received tofacitinib 10 mg b.i. d.

## Tofacitinib: efficacy in clinical studies

### Efficacy in OCTAVE Induction 1 and 2

In both induction studies, the proportion of patients who reached remission at week 8, the primary endpoint, was significantly higher under tofacitinib 10 mg b.i. d. than under placebo. Patients given tofacitinib 10 mg b.i. d. also achieved mucosal healing and other secondary endpoints significantly more frequently than those under placebo (Table 3; [5]).

**Table 3:** OCTAVE Induction 1 and 2: proportion of patients having achieved the efficacy endpoints at week 8 [5]

Endpoints^a^	OCTAVE Induction 1			OCTAVE Induction 2		
	Placebo (*n* = 122) (in %)	Tofacitinib 10 mg b.i. d. (*n* = 476) (in %)	*P*	Placebo (*n* = 112) (in %)	Tofacitinib 10 mg b.i. d. (*n* = 429) (in %)	*P*
*Remission*	8	19	0.007	4	17	< 0.001
*Mucosal healing*	16	31	< 0.001	12	28	< 0.001
*Clinical response*	33	60	< 0.001	29	55	< 0.001
*Endoscopic remission*	2	7	0.04	2	7	0.04

^a^For definitions, see Table 1

#### Recommendation 1:

The recommended tofacitinib dosage in UC induction treatment is 10 mg b.i. d. over 8 weeks.

#### Rapid onset of tofacitinib effect

Tofacitinib appears to become effective rapidly. According to a post hoc analysis of OCTAVE Induction 1 and 2, a significant improvement in stool frequency and rectal bleeding was already ascertained in the first days of treatment. After 3 days, the proportion of patients having experienced a decrease in the Mayo stool frequency subscore ≥ 1 was significantly higher under tofacitinib than under placebo (29% vs. 18%; *P* < 0.01) [6]. Likewise, after 3 days, the proportion of patients having experienced a decrease in the Mayo rectal bleeding subscore was also significantly higher under tofacitinib than under placebo (32% vs. 20%; *P* < 0.01) [6]. In both cases, this significant difference increased by the end of 8‑week induction therapy.

### Pretreatment with TNF-alpha inhibitors

In both subgroups with and without TNF-alpha inhibitor pretreatment, more patients under tofacitinib 10 mg b.i. d. achieved remission and/or mucosal healing at week 8 than under placebo (Table 4). The differences between tofacitinib and placebo were largely consistent in both subgroups.

**Table 4:** OCTAVE Induction 1 and 2: proportion of patients having reached the primary endpoint and the key secondary endpoint at week 8 after failure of TNF-alpha inhibitor treatment [1]

*Endpoints*^a^ Subgroup	OCTAVE Induction 1		OCTAVE Induction 2	
	Placebo (*n* = 122)	Tofacitinib 10 mg b.i. d. (*n* = 476)	Placebo (*n* = 112)	Tofacitinib 10 mg b.i. d. (*n* = 429)
*Remission*				
Previous TNF-alpha inhibitor failure	1/64 (2%)	27/243 (11%)	0/60 (0%)	26/222 (12%)
TNF‑alpha inhibitor naïve	9/58 (16%)	61/233 (26%)	4/52 (8%)	45/207 (22%)
*Mucosal healing*				
Previous TNF‑alpha inhibitor failure	4/64 (6%)	55/243 (23%)	4/60 (7%)	48/222 (22%)
TNF‑alpha inhibitor naïve	15/58 (26%)	94/233 (40%)	9/52 (17%)	74/207 (36%)

^a^For definitions, see Table 1

At week 52, similar superiority over placebo was seen with either dosage of tofacitinib in both subgroups with and without TNF-alpha inhibitor pretreatment [7].

### Non-response at week 8 of induction treatment

Patients in OCTAVE Induction 1 and 2, who had shown no response following 8 weeks of induction treatment, were eligible for participation in the phase 3 long-term OCTAVE Open continuation study, in which they were treated with tofacitinib 10 mg b.i. d. Those who continued to show no response after another 8 weeks of induction treatment were excluded from the study as per protocol. Overall, 52% of the patients, who had failed to clinically respond to tofacitinib 10 mg b.i. d. at week 8, achieved clinical response after 8 additional weeks of induction therapy. At month 12, 70% and 57% of these patients still achieved clinical response and mucosal healing, respectively, and 45% were in remission. By month 36, the proportions of these patients were 56%, 52% and 45%, respectively [8].

#### Recommendation 2:

In patients, in whom no sufficient treatment success is determined by week 8 of induction treatment with tofacitinib 10 mg b.i. d., the induction dose of 10 mg b.i. d. can be extended by another 8 weeks (i.e. 16 weeks in total).

#### Recommendation 3:

Treatment success and decisions regarding continued or discontinued tofacitinib therapy should be evaluated after 8–16 weeks. In the event of treatment response and concomitant corticosteroid treatment, cortisone is to be gradually reduced.

### Efficacy in OCTAVE sustain

Patients showing clinical response following 8 weeks of induction therapy in OCTAVE Induction 1 and 2 were eligible for participation in the phase 3 OCTAVE Sustain study [5]. At maintenance week 52, OCTAVE Sustain demonstrated significantly higher remission rates under both tofacitinib 5 mg b.i. d. and 10 mg b.i. d. (Table 5; [5]).

**Table 5:** OCTAVE Sustain: proportion of patients having achieved the efficacy endpoints at week 52 [1, 5]

Endpoints^a^	OCTAVE Sustain				
	Placebo (*n* = 198) (in %)	Tofacitinib 5 mg b.i. d. (*n* = 198) (in %)	*P*^c^	Tofacitinib 10 mg b.i. d. (*n* = 197) (in %)	*P*
*Remission*	11	34	< 0.001	41	< 0.001
*Mucosal healing*	13	37	< 0.001	46	< 0.001
*Sustained corticosteroid-free remission*	5	35	< 0.001	47	< 0.001
*Sustained remission*	10	22	< 0.001	25	< 0.001
*Clinical response*	20	52	< 0.001	62	< 0.001
*Endoscopic remission*^b^ *[* 1*]*	4	15	< 0.001	17	< 0.001

^a^For definitions, see Table 1

^b^Tofacitinib (Xeljanz®) prescribing information

^c^Vs. placebo

As had been the case in the induction studies, a larger proportion of OCTAVE Sustain patients under tofacitinib 5 mg b.i. d. and 10 mg b.i. d. than under placebo experienced remission, mucosal healing by week 52 in both subgroups with or without previous TNF-alpha inhibitor treatment failure (Table 6; [5]).

**Table 6:** OCTAVE Sustain: proportion of patients having achieved the primary and important secondary endpoints at week 52 according to TNF-alpha inhibitor subgroups [1]

Endpoints^a^	Placebo (*n* = 198)	Tofacitinib 5 mg b.i. d. (*n* = 198)	Tofacitinib 10 mg b.i. d. (*n* = 197)
*Remission*			
Previous TNF‑α inhibitor failure	10/89 (11%)	20/83 (24%)	34/93 (37%)
TNF‑α inhibitor-naïve	12/109 (11%)	48/115 (42%)	46/104 (44%)
*Mucosal healing*			
Previous TNF‑α inhibitor failure	11/89 (12%)	25/83 (30%)	37/93 (40%)
TNF‑α inhibitor-naïve	15/109 (14%)	49/115 (43%)	53/104 (51%)

^a^For definitions, see Table 1

In the subgroup of patients without previous TNF-alpha inhibitor failure the treatment difference vs. placebo was similar under tofacitinib 5 mg b.i. d. and 10 mg b.i. d. In the subgroup of patients with previous TNF-alpha inhibitor failure the treatment difference vs. placebo was 10–17% larger under tofacitinib 10 mg b.i. d. than under tofacitinib 5 mg b.i.d with regard to the primary and most important secondary endpoint (Table 6; [5]).

### Efficacy in OCTAVE Open

OCTAVE Open included more than 1000 subjects from various cohorts of the OCTAVE precursor studies. OCTAVE Sustain patients in remission at week 52 were further treated with tofacitinib 5 mg b.i. d. in this long-term study, all others received tofacitinib 10 mg b.i. d. (Fig. 1).

#### Sustained response in long-term treatment

The cohort given remission maintenance therapy comprised the OCTAVE Sustain patients in remission at week 52 (*n* = 175), who were further treated with tofacitinib 5 mg b.i. d. in OCTAVE Open. Subjects experiencing exacerbation and/or a dose increase due to an ongoing inflammation were excluded from the analysis. In OCTAVE Open, half of the patients in remission at month 12 of tofacitinib maintenance therapy were still in remission after 36 months (Table 7). Thus, half of the patients experienced sustained efficacy after a total of 4 years of tofacitinib treatment [9].

**Table 7:** Proportion of patients in remission at week 52 in OCTAVE Sustain with sustained efficacy at months 2, 12, 24 and 36 under tofacitinib 5 mg b.i. d. in OCTAVE Open [9]

Endpoints^a^	Month 2	Month 12	Month 24	Month 36
Remission	131/175 (75%)	129/175 (74%)	103/175 (59%)	103/175 (59%)
Mucosal healing	152/175 (87%)	140/175 (80%)	119/175 (68%)	113/175 (65%)
Clinical response	159/175 (91%)	147/175 (84%)	125/175 (71%)	117/175 (67%)

^a^For definitions, see Table 1

##### Recommendation 4:

Maintenance therapy with tofacitinib 10 mg b.i. d. is to be as short as possible. In the event of clinical and biomarker remission under 10 mg b.i. d., the attempt should be made to reduce the dosage to 5 mg b.i. d.

#### Improvement of clinical response in long-term treatment

Patients having achieved clinical response but no remission by week 52 in OCTAVE Sustain were further treated with tofacitinib 10 mg b.i. d. in OCTAVE Open (*n* = 82) (Fig. 1 and Table 1). Overall, 70% (41/59) of the patients were able to maintain clinical response until month 24 in this long-term trial. At month 2 in OCTAVE Open, 59% (48/82) of the patients improved from clinical response to remission and 58% of these patients were still in remission after 24 months [10].

#### Dose escalation in OCTAVE Open after loss of response in OCTAVE Sustain

The dose escalation cohort comprised the patients having received tofacitinib 5 mg b.i. d. in OCTAVE Sustain and having shown loss of response in the course of the 52-week maintenance treatment. These subjects (*n* = 58) were further treated with tofacitinib 10 mg b.i. d. in OCTAVE Open. Approximately one third of the patients already experienced remission and more than half achieved clinical response by month 2 following dose escalation to 10 mg b.i. d. By month 36, the proportion of patients with remission, mucosal healing and clinical response was maintained and/or expanded [11].

##### Recommendation 5:

A tofacitinib dose increase to 10 mg b.i. d. can improve response in patients who lost response after switching to maintenance therapy with tofacitinib 5 mg b.i. d.

In the presence of known risk factors for venous thrombosis (VT), patients should not be switched to 10 mg b.i. d. as long as there are treatment alternatives (see also Recommendation 13).

#### Time to loss of efficacy after treatment discontinuation and response after treatment resumption

A post hoc analysis examined the treatment discontinuation cohort (*n* = 174), which had achieved clinical response after 8 weeks of 10 mg b.i. d. induction treatment in OCTAVE Induction 1 and 2 and was subsequently randomized to the placebo group in the OCTAVE Sustain maintenance study [12].

The time to loss of efficacy in OCTAVE Sustain and the response rate after resuming treatment in OCTAVE Open were analyzed in this cohort [13]. Symptom recurrence was observed in 75% of the patients by the end of the 52-week maintenance phase in OCTAVE Sustain. The median time to loss of efficacy was 135 days (interquartile range, 63–371 days). After treatment resumption, 32% (56/174) of the patients having experienced loss of efficacy re-achieved clinical response, 16% (27/174) mucosal healing and 16% (27/174) remission after 24 weeks. By week 52, the rates of clinical response, remission and mucosal healing were 19% (33/174), 10% (18/174) and 13% (22/174), respectively [13].

##### Recommendation 6:

Renewed induction treatment with tofacitinib 10 mg b.i. d. can be given in the event of treatment discontinuation and loss of response.

## Tofacitinib: safety in clinical studies

### General safety profile

Various patient cohorts were defined in order to analyze the safety data of tofacitinib. The induction cohort comprised the patients in the phase 2 and phase 3 induction studies. The maintenance cohort included the subjects treated with tofacitinib 5 mg b.i. d., tofacitinib 10 mg b.i. d. or placebo in OCTAVE Sustain. The overall cohort comprised patients treated with 5 mg b.i. d. or 10 mg b.i. d. in phase 2, phase 3 or the long-term OCTAVE Open study and thus facilitated an integrated safety analysis across the entire development program in UC.

The overall cohort comprised 944 patients with 6 years of median treatment and 2400.8 patient years (PYs). The majority of these patients (81%) had been treated with tofacitinib 10 mg b.i. d. throughout most of the treatment [9].

The frequency of adverse events (AEs) and serious AEs (SAEs) was comparable in all groups of the maintenance cohort (Table 8).

**Table 8:** Tofacitinib in ulcerative colitis: Safety in the maintenance and overall cohorts of the tofacitinib clinical development program [9, 14]

	Maintenance cohort			Overall cohort
	Placebo (*n* = 198)	Tofacitinib 5 mg b.i. d. (*n* = 198)	Tofacitinib 10 mg b.i.d (*n* = 196)	All tofacitinib (*n* = 944)
*Exposure in PYs*	100	146	154	2441
*Duration of treatment in days, median (range)*	138 (14–382)	364 (22–420)	368 (1–399)	1529 (36–2422)
*Patients with AEs, n (%)*	149 (75)	143 (72)	156 (80)	780 (83)
*Patients with SAEs, n (%)*	13 (7)	10 (5)	11 (6)	186 (20)

*AE* adverse event, *PY* patient year, *SAE* serious adverse event

### AEs of special interest

The incidence rates (IRs) of individual safety events of special interest per 100 PYs in the maintenance and overall cohorts are summarized in Table 9.

**Table 9:** Tofacitinib in ulcerative colitis: AEs of special interest in the maintenance and overall cohorts of the tofacitinib development program [9]

	Maintenance cohort					Overall cohort	
	Tofacitinib 5 mg b.i. d. (*n* = 175)			Tofacitinib 10 mg b.i. d. (*n* = 769)		Per 2022 (*n* = 944)	
	n (%)	IR (95% CI)		n (%)	IR (95% CI)	n (%)	IR (95% CI)
**Infections**							
*SIs*	8 (5)	1.25 (0.54–2.46)		31 (4)	1.74 (1.18–2.47)	39 (4)	1.61 (1.14–2.20)
*Opportunistic infections*^*a,b*^	4 (2)	0.63 (0.17–1.60)	17 (2)	0.96 (0.56–1.53)	21 (2)	0.87 (0.54–1.33)	
*HZ infections*	13 (7)	2.8 (1.11–3.55)	60 (8)	3.55 (2.71–4.58)	73 (8)	3.16 (2.47–3.97)	
**Malignoma**							
*Malignoma, excluding non-melanoma skin cancer*^a^	7 (4)	1.09 (0.44–2.25)		18 (2)	1.0 (0.60–1.59)	25 (3)	1.03 (0.67–1.52)
*Non-melanoma skin cancer*^a^	6 (3)	0.96 (0.35–2.08)		12 (2)	0.68 (0.35–1.19)	18 (2)	0.75 (0.45–1.52)
**Cardiovascular events**							
*MACEs*^a^	2 (1)	0.31 (0.04–1.13)		2 (0)	0.11 (0.01–0.4)	4 (0)	0.16 (0.04–1.19)
*Deep vein thrombosis*	0 (0)	0.0 (0.0–0.57)		1 (0)	0.06 (0.0–0.31)	1 (0)	0.04 (0.0–0.23)
*Pulmonary embolism*	0 (0)	0.0 (0.0–0.57)		5 (1)	0.28 (0.09–0.65)	5 (1)	0.21 (0.7–0.48)
*Gastrointestinal tract perforation*^a^	1 (1)	0.16 (0.0–0.87)		1 (0)	0.06 (0.0–0.31)	2 (0)	0.08 (0.01–0.3)
*Deaths*^c^	0 (0)	0.0 (0.0–0.57)		6 (1)	0.33 (0.12–0.73)	6 (1)	0.25 (0.09–0.54)

*CI* confidence interval, *HZ* herpes zoster, *IR* incidence rate per 100 patient years, *MACE* major adverse cardiovascular event, *SI* severe infection, *CI* confidence interval

^a^Adjudged events (data from phase 2 study not included in the overall cohort)

^b^Exclusively tuberculosis and herpes zoster with 2 adjacent dermatomes

^c^All deaths were not related to tofacitinib

In essence, a comparison of the safety data over time yielded consistent event rates (Table 9). The safety profile of tofacitinib from the OCTAVE approval studies for UC was consistent with that shown in the approval studies for RA and PsA [1].

### Severe infections

In general, tofacitinib is associated with an increased risk of severe infections (SIs). The IR of SIs in the overall cohort was 1.61/100 PYs. The risk of SIs seems to be unrelated to the administered dosage and was mainly identified in the induction phase with 10 mg b.i. d. [9].

#### Recommendation 7:

The risk-benefit profile of tofacitinib treatment is to be carefully examined in older patients and those at an increased risk of infection, e.g., diabetes mellitus.

See the information issued by the Federal Office of Public Health (Bundesamt für Gesundheitswesen) regarding the safety of tofacitinib, 8 July 2021 [15].

#### Tuberculosis

In patients having been exposed to tuberculosis or having visited regions affected by endemic tuberculosis, the risks and benefits associated with tofacitinib are to be considered prior to treatment. In accordance with current guidelines, and by analogy with TNF-alpha inhibitor treatment, patients are to be examined for latent or active tuberculosis prior to and in the course of tofacitinib administration [1].

Active tuberculosis is a contraindication to the application of tofacitinib. Patients with latent tuberculosis, who are tested positive, are to be treated with antimycobacterial standard treatment prior to tofacitinib intake.

#### Opportunistic infections

The IR for opportunistic infections has recently been reported as 0.87/100 PYs and appears to be higher with a dosage of 10 mg b.i. d. than with 5 mg b.i. d. (Table 9; [13]). The risk was dose-dependent, but unrelated to the duration of treatment.

##### Recommendation 8:

Opportunistic infections under tofacitinib are possible. In the event of pneumonia, consideration should be given to the presence of atypical pathogens.

#### Herpes zoster infections

The IR of herpes zoster (HZ) infections has been shown to be dose-dependent. Amounting to 3.16/100 PYs with all tofacitinib dosages, [9] it was slightly higher than the IR seen with immunosuppressives and biologics [16, 17]. The HZ risk was unrelated to the duration of treatment.

Advanced age (≥ 65 years), preceding TNF-alpha inhibitor administration, corticosteroid treatment at baseline and Asian origin increased the IR of HZ [18]. HZ infections were identified in 73 patients (8%) under tofacitinib in the overall cohort (*n* = 944) and 7 (10%) of these 73 cases were severe cases, 12 patients had HZ infections with 2 adjacent dermatomes, 12 were multidermatomal and 5 disseminated [9].

##### Recommendation 9:

As tofacitinib treatment leads to a dose-dependent increase in the risk for HZ, a prophylactic vaccination is recommended.

#### Vaccinations

Prior to tofacitinib treatment, all patients’ immunization statuses are to be updated in accordance with the current vaccination recommendations [1, 19]. When deciding on the application of live-attenuated vaccines prior to tofacitinib treatment, the given patient’s pre-existing immunosuppression should be considered. No data are available concerning the secondary transmission of infections by way of live-attenuated vaccines to patients under tofacitinib.

##### Recommendation 10:

Shingrix®, the first inactivated vaccine against HZ infections, was approved in Europe in 2018 [20]. Serological testing for anti-varicella zoster virus (VZV) antibodies is not required when applying this vaccine. Shingrix® can also be applied under ongoing tofacitinib treatment.

The live-attenuated HZ vaccine Zostavax® is to be exclusively administered to patients with a history of varicella or seropositive VZV findings. In the event of uncertain varicella status, an anti-VZV antibody test is to be carried out. Zostavax® is contraindicated in immunodeficiency [20].

##### Recommendation 11:

Live-attenuated vaccines are not to be applied concomitantly with tofacitinib [1]. These vaccines are to be given at least 4 weeks prior to tofacitinib treatment or in accordance with the current vaccination recommendations regarding the administration of immunomodulating agents.

#### Hepatitis

Based on currently available data on the effect of tofacitinib on the reactivation of chronic viral hepatitis, the risk is to be assessed as low to moderate, whereas the data refer to patients with RA [21, 22].

Patients tested positive for hepatitis B or C were excluded from participation in the clinical studies. Prior to tofacitinib treatment, patients should be examined for viral hepatitis and measures taken in accordance with those applicable for TNF-alpha inhibitors.

### Malignoma

The effect of tofacitinib on the genesis and etiopathology of malignoma is not known. An IR of 1.03/100 PYs for malignoma (excluding non-melanoma skin cancer) was ascertained on the basis of the OCTAVE Open study (Table 9; [9]). Overall, malignant disease (excluding non-melanoma skin cancer) was seen in 25 of 944 patients, of whom most had been treated with tofacitinib 10 mg b.i. d.

A recently published study in patients with RA on the risk of cardiovascular and malignant diseases demonstrated that the incidence of malignoma (excluding non-melanoma skin cancer) under tofacitinib is increased as compared to TNF-alpha inhibitor treatment regardless of tofacitinib dosage (IR: 1.13 vs. 0.77); however, it is important to note the RA patients included in that study were older than 50 years and showed at least one cardiovascular risk factor [23].

#### Recommendation 12:

In patients with a previous cancerous condition (excluding successfully treated non-melanoma skin cancer), the risks and benefits of tofacitinib treatment are to be considered prior to treatment. Patients under ongoing treatment for malignant disease are not to be treated with tofacitinib.

### Non-melanoma skin cancer

Cases of non-melanoma skin cancer have also been detected in patients under tofacitinib. In view of the particular risk factors for non-melanoma skin cancer (e.g., administered pretreatment), this tumor entity is referred to separately. In the overall cohort, the IR was recently seen to amount to 0.75/100 PYs (Table 9; [9]). A dose-dependent risk was not ruled out.

### Major adverse cardiovascular events

Major adverse cardiovascular events (MACEs) comprised cardiovascular deaths, non-fatal myocardial infarction and non-fatal stroke. In the final overall cohort, the MACE IR with 4 cases amounted to 0.16/100 PYs (Table 9; [9]). The IR of MACEs in a similar cohort of UC patients under TNF-alpha inhibitors (infliximab, adalimumab and golimumab) was 0.51/100 PYs (95% CI 0.31–0.79). [24] The occurrence of MACEs under tofacitinib was unrelated to dosage.

In contrast, a recently published study by Ytterberg et al. showed an increased risk of MACEs under tofacitinib as compared to TNF-alpha inhibitors in patients with RA [25].

### Venous thromboembolic events (VTEs) under tofacitinib

#### VTE risk under tofacitinib in RA

Within the framework of an interim analysis of the post-marketing ORAL Surveillance study in patients with RA, who were 50 years of age or older and presented with at least one cardiovascular risk factor, those under tofacitinib 10 mg b.i. d. were seen to be at increased risk of pulmonary embolism and all-cause mortality as compared to those given TNF-alpha inhibitors (Table 10; [1, 22, 23]).

**Table 10:** Incidence of venous and arterial thromboembolic event in tofacitinib clinical study programs in RA, PsA and PsO and in non-interventional observational studies [23]

Indication	Deep vein thrombosis IR (95% CI)		Pulmonary embolism IR (95% CI)		Arterial thromboembolic event IR (95% CI)	
	Tofacitinib 5 mg b.i. d.	Tofacitinib 10 mg b.i. d.	Tofacitinib 5 mg b.i. d.	Tofacitinib 10 mg b.i. d.	Tofacitinib 5 mg b.i. d.	Tofacitinib 10 mg b.i. d.
*RA (n = 7964)*	0.17 (0.09–0.27)	0.15 (0.09–0.22)	0.12 (0.06–0.22)	0.13 (0.08–0.21)	0.32 (0.22–0.46)	0.38 (0.28–0.49)
*PsO (n = 3663)*^a^	0.06 (0.00–0.36)	0.06 (0.02–0.15)	0.13 (0.02–0.47)	0.09 (0.04–0.19)	0.52 (0.22–1.02)	0.22 (0.13–0.35)
*PsA (n = 783)*	0.00 (0.00–0.28)	0.13 (0.00–0.70)	0.08 (0.00–0.43)	0.00 (0.00–0.46)	0.31 (0.08–0.79)	0.38 (0.08–1.11)

*CI* confidence interval, *IR* incidence rate per 100 patient years, *PsA* psoriatic arthritis, *PsO* psoriasis, *RA* rheumatoid arthritis

^a^Note: tofacitinib is not approved for the treatment of PsO

The IRs of all examined events were dose-independent across the clinical study programs in RA, PsO and PsA. Moreover, the IRs assessed in the clinical studies were consistent with the results of non-interventional studies and the IRs published for comparable treatments [23].

As anticipated, the IRs of thromboembolic events were higher in patients with corresponding risk factors than in those without those risk factors and they were consistent with the results of the ORAL Surveillance analysis [23].

#### VTE risk under tofacitinib in ulcerative colitis

The final analysis of the OCTAVE Open study yielded a VTE risk with an IR of 0.04/100 PYs for the overall cohort ([9]; Table 9). In a recently published safety profile study in patients with RA, an increased risk of VTEs was seen in subjects treated with tofacitinib than in those given TNF inhibitors; however, it is important to note that this applied exclusively to patients beyond the age of 50 years, who presented with at least one cardiovascular risk factor. Thus, the increase in the risk of VTEs was evidenced for RA, yet not for UC [9, 22].

##### Recommendation 13:

In patients with UC and known risk factors for VTEs, tofacitinib is to be applied with caution regardless of dosage (see also Recommendations 4 and 5). Over the course of tofacitinib treatment, patients are to be informed upfront and regularly about changes and early symptoms, such as swelling, pressure pain, etc., of the VTE risk.

### Mortality

A total of 6 deaths were registered in the course of the OCTAVE study programme (aortic dissection, hepatic angiosarcoma, acute myeloid leukemia, pulmonary embolism as a complication of a metastasized cholangiocarcinoma, malignant melanoma) (Table 9; [9]) of which 1 occurred in the induction cohort and 5 in OCTAVE Open. The IR for the final overall cohort was 0.25 (95% CI 0.09–0.54) [9].

## Treatment management

### Monitoring of laboratory parameters

#### Lymphocytes, neutrophils and hemoglobin

Patients under tofacitinib in controlled clinical studies have been seen to more frequently experience lymphopenia, neutropenia and anemia. Treatment with tofacitinib is not to be initialized in patients with an absolute lymphocyte count of < 750 cells/mm^3^, an absolute neutrophil count of < 1000 cells/mm^3^ or a hemoglobin value of < 9 g/dl. [1].

##### Recommendation 14:

Lymphocytes, neutrophils and hemoglobin are to be controlled at baseline, after week 2 and week 6 of treatment and subsequently every 3 months. In dose-related abnormal laboratory results, such as lymphopenia, neutropenia and anemia, dose reduction or treatment discontinuation may become necessary.

### Lipid values

While treatment with tofacitinib has been shown to result in a moderate and reversible increase in lipid levels, the low-density lipoprotein : high-density lipoprotein (LDL:HDL) and the total cholesterol:LDL ratios remained unchanged [25]. In general, maximum effects were observed within 6 weeks.

#### Recommendation 15:

Blood lipid levels are to be examined at certain time points after beginning treatment with tofacitinib. Statin treatment can be applied to lower elevated total cholesterol and LDL values in connection with tofacitinib to pretreatment levels [1].

### Hepatic and renal function

The clearance mechanisms for tofacitinib are approximately 70% hepatic metabolism and 30% renal excretion. In limited hepatic function, the dosage is to be reduced in accordance with the degree of impairment.

#### Recommendation 16:

No dose adaptation of tofacitinib is required in mild and moderate renal dysfunction and the daily dosage is to be halved in severe kidney impairment. In patients requiring dialysis, the dosage is to remain reduced after hemodialysis [1]. No dose adaptation is required in mild hepatic dysfunction (Child-Pugh score A) and the daily dosage is to be halved in moderate liver impairment (Child-Pugh score B). Tofacitinib is not to be applied in severe hepatic dysfunction (Child-Pugh score C).

### Pregnancy and lactation

No well-controlled studies are available with respect to tofacitinib administration in pregnant women [1]. The ability of tofacitinib to pass the placental barrier has not been studied, yet placental transfer is to be assumed due to its low molecular weight [26].

Applied in rat and rabbit models at dosages many times higher than those used in humans, tofacitinib had a teratogenic effect and detrimentally affected perinatal and postnatal development. As it is not known whether tofacitinib secretes into human breast milk, a risk of exposure to tofacitinib for breastfed children cannot be ruled out. Tofacitinib has been seen to excrete into the milk of lactating rats. Therefore, according to prescribing information, its administration during lactation is contraindicated. Tofacitinib was observed to impair female, but not male, fertility in rat models. No clinical studies have been carried out on the potential effect of tofacitinib on human fertility [1].

In a register of births recording pregnancies under tofacitinib, there were 301 UC patients of childbearing age among the 1157 subjects in the overall cohort. Of these UC patients of childbearing age, 11 pregnancies showed maternal and 14 pregnancies showed paternal tofacitinib exposure. Of the 19 pregnancies with known outcomes, 15 resulted in healthy children (including 1 preterm delivery), 2 in spontaneous miscarriage and 2 in medically indicated termination. There were no cases of fetal deaths, neonatal deaths or congenital deformities [27].

#### Recommendation 17:

The incidence of congenital deformities and spontaneous miscarriages in clinical studies with tofacitinib seems to be largely consistent with the background risk in the normal population; however, in view of the results of preclinical studies, the lack of controlled clinical studies and the scarcity of prospective registry data, patients under ongoing tofacitinib treatment are advised against pregnancy. Women of childbearing age are to apply a reliable method of contraception during their tofacitinib treatment and for at least 4 weeks after their last intake of tofacitinib.

## Real-world experiences

### Efficacy in clinical practice

Apart from clinical approval studies, the number of publications on the efficacy of tofacitinib has steadily grown and a meta-analysis of published real-world experiences in the application of tofacitinib has been released; however, due to the variety of designs, dosages and endpoints, it is at times difficult to directly compare those investigations. In non-controlled real-world studies, the initial dosage of tofacitinib applied in induction was either 5 mg b.i. d. (off label) or 10 mg b.i. d. with the option to increase the dosage in the absence of efficacy. An intermittent dose increase was also applied over a long-term course with a 50% success rate and, if well tolerated, was to be attempted prior to definitive diagnosis of treatment failure. In the case of high tolerability and efficacy, both 5 mg b.i. d. and 10 mg b.i. d. were to be used as dosages after induction. The currently largest published meta-analysis comprised 17 studies with a total of 1162 UC patients [28].

In an average of 62% of the patients, this meta-analysis showed clinical response at week 8, which remained stable from week 12 to week 16 at 64%. As was the case in the clinical OCTAVE study program, patients having received pretreatment with biologics showed a slightly lower response rate than biologic-naïve patients. Clinical response was still seen in 51% and 42% of the subjects by months 6 and 8, respectively. On average, 35% of the patients achieved clinical remission by week 8 and 47% from week 12 to week 16. After 12 months, the clinical remission rate was still 41%. Steroid-free clinical remission was reached by 38% of the patients at week 8 and by 44% between weeks 12 and 16. By month 6 and month 12, 34% and 31% of the subjects, respectively, were still in steroid-free clinical remission. Mucosal healing was achieved in 42% of the patients by week 8 and in 35% from week 12 to week 16.

The data from the approval studies (OCTAVE study program) were markedly different from the real-world data in terms of safety and side effect documentation. While the approval studies reported AE rates of 72–87%, the side effect percentages in the real-world data were 16–27% [27, 29]. It is important to note that the most frequent real-world AEs were infections, which were identified in 5–20% of the patients.

Pre-existing conditions and concomitant immunosuppressive treatment, particularly with steroids, appear to increase the risk of infections. In all studies, a slight increase in cholesterol was identified, which, however, did not make specific measures necessary. Long-term effects on cardiovascular disorders are not known. Cases of HZ were seen in approximately 2% of the patients. No thromboembolic events were observed in the time period. One expected death (a patient with known gallbladder cancer) was recorded. In a cohort study with 260 patients treated at 6 centers in the USA and 6 months of median follow-up, side effects—most of which (5%) were infections—were identified in 16% of the patients. Initial meta-analyses of the safety of JAK inhibitors again yielded an increased HZ risk, yet there was no increase in other side effects [30, 31].

A meta-analysis published by Taxonera et al. examined real-world experiences with regard to the efficacy and safety of tofacitinib in 17 studies including 1162 patients with UC [28]. This meta-analysis corroborated the mentioned results and showed no increased risk of side effects, cardiovascular events or thromboembolic events [32]. Patients under tofacitinib are to be comprehensively informed about the risks and benefits associated with this treatment.

#### Recommendation 18:

The real-world data are consistent with the available data from approval studies with regard to the efficacy and safety of tofacitinib in patients with UC. Furthermore, the real-world data do not demonstrate an increased risk of cardiovascular and thromboembolic events.

### Tofacitinib and COVID-19 in patients with ulcerative colitis

Recent data suggest that tofacitinib treatment does not pose an increased risk of a coronavirus disease 2019 (COVID-19) infection or a more severe course of infection in patients with ulcerative colitis. There are also data that tofacitinib can presently even be applied to prevent possible severe courses of COVID-19 infections. There is no reason, therefore, to postpone planned induction of or to interrupt ongoing treatment with tofacitinib [33, 34].

#### Recommendation 19:

Tofacitinib does not imply an increased risk of COVID-19 infection or severe courses in the event of infection. Planned tofacitinib therapy is not to be postponed and ongoing treatment is not to be interrupted.

## Conclusion

Based on the data currently available for tofacitinib, its oral application, fast-acting and good efficacy—also in patients who had a previous failure of biological treatment—individual dosing options, good manageability due to its short half-life and absence of immunogenicity altogether militate in favor of this immunomodulating agent. Particularly in biologic pretreated UC patients younger than 60 years, who show a high level of disease activity and require rapid efficacy, tofacitinib proves to be a valuable treatment option—with due regard to the known risk factors and contraindications.

This position paper does not aim to compare the efficacy and safety issues of tofacitinib with other advanced targeted therapies for the treatment of ulcerative colitis.
